# The Role of Palliative Care in Chronic Progressive Neurological Diseases—A Survey Amongst Neurologists in the Netherlands

**DOI:** 10.3389/fneur.2018.01157

**Published:** 2019-01-14

**Authors:** Hannah A. W. Walter, Antje A. Seeber, Dick L. Willems, Marianne de Visser

**Affiliations:** ^1^Department of Neurology, Amsterdam University Medical Center, Academic Medical Center, University of Amsterdam, Amsterdam, Netherlands; ^2^Section of Medical Ethics, Department of General Practice, Amsterdam University Medical Center, Academic Medical Center, University of Amsterdam, Amsterdam, Netherlands

**Keywords:** palliative care, advance care planning, nervous system diseases, decision making, Parkinson's disease, multiple sclerosis, glioma

## Abstract

**Background:** Chronic progressive neurological diseases like high grade glioma (HGG), Parkinson's disease (PD), and multiple sclerosis (MS) are incurable, and associated with increasing disability including cognitive impairment, and reduced life expectancy. Patients with these diseases have complex care needs. Therefore, timely advance care planning (ACP) is required. Our aim was to investigate timing and content of discussions on treatment restrictions, i.e., to initiate, withhold, or withdraw treatment in patients with HGG, PD, and MS, from the neurologists' perspective.

**Methods:** We performed a national online survey amongst consultants in neurology and residents in The Netherlands. The questionnaire focused on their daily practice concerning timing and content of discussions on treatment restrictions with patients suffering from HGG, PD or MS. We also inquired about education and training in discussing these issues.

**Results:** A total of 125 respondents [89 neurologists (71%), 62% male, with a median age of 44 years, and 36 residents (29%), 31% male with a median age of 29 years] responded. Initial discussions on treatment restrictions were said to take place during the first year after diagnosis in 28% of patients with HGG, and commonly no earlier than in the terminal phase in patients with PD and MS. In all conditions, significant cognitive decline was the most important trigger to advance discussions, followed by physical decline, and initiation of the terminal phase. Most discussed issues included ventilation, resuscitation, and admission to the intensive care unit. More than half of the consultants in neurology and residents felt that they needed (more) education and training in having discussions on treatment restrictions.

**Conclusion:** In patients with HGG discussions on treatment restrictions are initiated earlier than in patients with PD or MS. However, in all three diseases these discussions usually take place when significant physical and cognitive decline has become apparent and commonly mark the initiation of end-of-life care. More than half of the responding consultants in neurology and residents feel the need for improvement of their skills in performing these discussions.

## Introduction

Chronic progressive neurological diseases like amyotrophic lateral sclerosis (ALS), high grade glioma (HGG), Parkinson's disease (PD), and multiple sclerosis (MS) are incurable and often associated with a shortened life expectancy. Patients with these diseases have a host of unmet physical, cognitive, psychosocial, and spiritual needs and experience problems in coordination and continuity of care (1, 2). There is growing evidence that early integration of palliative care improves the quality of life of these patients and their significant others. However, realization of this integration appears to be challenging in every day practice (3–8). The presence of communication barriers, e.g., speech impairment frequently observed in ALS and PD, and cognitive or behavioral disturbances as are found in HGG, PD, and MS complicate matters even more.

Misconceptions about palliative care are common, amongst health care professionals and patients. First, palliative care is often considered to be synonymous with hospice care or end-of-life care (9). Second, illness trajectories of progressive neurological diseases vary from rapidly progressive (ALS, HGG) to prolonged and fluctuating (PD, MS). Patients with these diseases have significantly different symptom profiles, psychosocial issues, and spiritual needs (1, 10, 11). Consequently, their caregivers' burden is equally variable. Third, knowledge about palliative care needs in chronic progressive neurological diseases is just emerging (3, 12, 13). Fourth, health care professionals in general are found to not be familiar with communication skills needed to deliver bad news and to discuss advance care planning (ACP) (14–19).

ACP is a communication process in which patients' wishes, preferences, and goals with regard to future (palliative) care, including end-of-life care, are discussed in a timely, and iterative manner (20, 21). ACP includes considerations about disease- and symptom-specific treatment, resuscitation and other life-prolonging modalities, treatment restrictions, end-of-life wishes and appointment of surrogate decision-makers. There is an increasing body of evidence, mostly from research in patients with cancer and other non-neurological chronic progressive diseases, that ACP improves both the quality of end-of-life care, as well as patient and family satisfaction, and may reduce stress, anxiety, and depression in surviving relatives (22, 23). In ALS, the paradigmatic disease for palliative care in neurodegenerative disorders, it is common knowledge that discussions about future care should be done in an ongoing, iterative way (2, 24, 25). There is sparse evidence that in patients who are severely ill after stroke or with dementia, ACP is restricted to discussions about the care in the last phase of life (26). The same applies to ACP in patients with HGG (12, 27). Whether and how ACP takes place in long-term follow-up of patients with PD and MS has not been investigated so far.

The objective of our study was 2-folded. First, we aimed to investigate timing and content of discussions on treatment restrictions, i.e., to initiate, withhold, or withdraw treatment in the course of HGG, PD, and MS, from the neurologist's perspective. The focus was on these three conditions because in the Netherlands neurologists generally are involved in the follow-up of patients with HGG, PD, and MS, whereas specialists of other disciplines take care of patients with ALS, dementia, and post-stroke sequelae. Second, we compared our results with international data about ACP in patients with ALS.

## Materials and Methods

### Study Design and Population

We conducted a national cross-sectional survey amongst consultants in neurology and residents in The Netherlands. For reasons of privacy, we approached the potential participants via the secretariat of the neurology departments with the request to provide the physicians with the link to the online questionnaire. In order to maximize the response rate, two reminders were sent within the following 3 months. Data collection started in February 2016 and ended in August 2016. The questionnaire focused on three progressive neurological diseases, i.e., HGG, PD, and MS.

### Ethics Approval

Dutch law specifies that ethics approval is only needed when ‘participants are subject to procedures or are required to follow rules of behavior' (http://www.ccmo.nl/en/your-research-does-it-fall-under-the-wmo). As this was not the case, written informed consent was not required from the participants, as confirmed in a letter from the AMC local research ethics committee (REC) from 11th October 2018. Participants knew that the received data would be treated confidentially and used anonymized only, and that they could withdraw from the study at any moment, without explanation.

### Survey Questionnaire

The questionnaire was designed by the authors, partly based on the literature and partly based on the results of in-depths interviews with neurologists by one of the authors (AAS) (28). The online tool Survey Monkey (www.surveymonkey.com) was used. The questionnaire consisted of 57 questions, subdivided into three different sections. In the first section, questions were raised about the experiences of neurologists and residents with timing and content of discussions on potential treatment restrictions held with patients suffering from HGG, PD, and MS. Actually, we used the terms “considerations,” “initiating,” “withholding,” and/or “withdrawing,” and “common/accepted treatment option.” In the second section, neurologists were asked to elaborate on a recent case of HGG, PD, or MS in which such discussions took place. In the third section, there were questions on education and training in communicating treatment restrictions with patients and families. The questions were either pre-structured or open-ended. Two pilots were done amongst 10 neurologists and the questionnaire was adjusted according to their feedback.

### Analysis

Data analysis was conducted by SPSS (IBM Corp. Released 2016. IBM SPSS Statistics for Windows, Version 24.0. Armonk, NY: IBM Corp). Frequencies and proportions were calculated by descriptive statistics for categorical variables. Mean and standard deviation and median and range were calculated for continuous variables. Open-ended questions were analyzed and coded by three authors (HAWW, AAS, MdV).

## Results

There were 991 consultants and 341 residents who were contacted via their medical secretariats. One hundred twenty-five of them responded to the online survey, 89 (71%) consultants and 36 (29%) residents, from 63 hospitals (out of 79), yielding an overall response rate of 15% participants, but a response rate of 80% neurology practices. A total of 72 (58%) respondents (58 neurologists and 14 residents) filled in the survey completely. Therefore, the data was analyzed with a varying number of missing values.

### Profile of Respondents

Table 1 shows the demographics of the respondents. Amongst consultants in neurology, 48 (54%) worked as general neurologist, 35 (39%) also had a subspecialty area, and 41 (46%) worked in a subspecialty area only. Twenty (22%) neurologists had specific expertise in movement disorders, 18 (20%) in neuro-oncology and 19 (19%) in MS. Within the group of residents, 25 (75%) worked in general neurology, and 15 (44%) also worked in a subspecialty area. Nine residents (26%) worked in a subspecialty niche only. Eight (22%) residents had specific expertise in neuromuscular diseases, 4 (12%) in movement disorders, 3 (9%) in neuro-oncology, 1 (3%) in MS, and 6 (18%) in vascular neurology.

**Table 1:** Demographics of consultants in neurology and residents.

		**Consultant** ***n* (%)**		**Resident** ***n* (%)**
n (% male/ % female)		89 (62/38)		36 (31/69)
Age in years (median, range) (*n* = 61)		44 (33–64)		29 (25–40)
Number of working years	0–5	21 (24)	0–2	15 (42)
	5–10	23 (26)	2–4	9 (25)
	10–15	17 (19)	4–6^*^	12 (33)
	>15	28 (31)	

* *In The Netherlands, the duration of neurological training is 6 years*.

**Figure 1:**
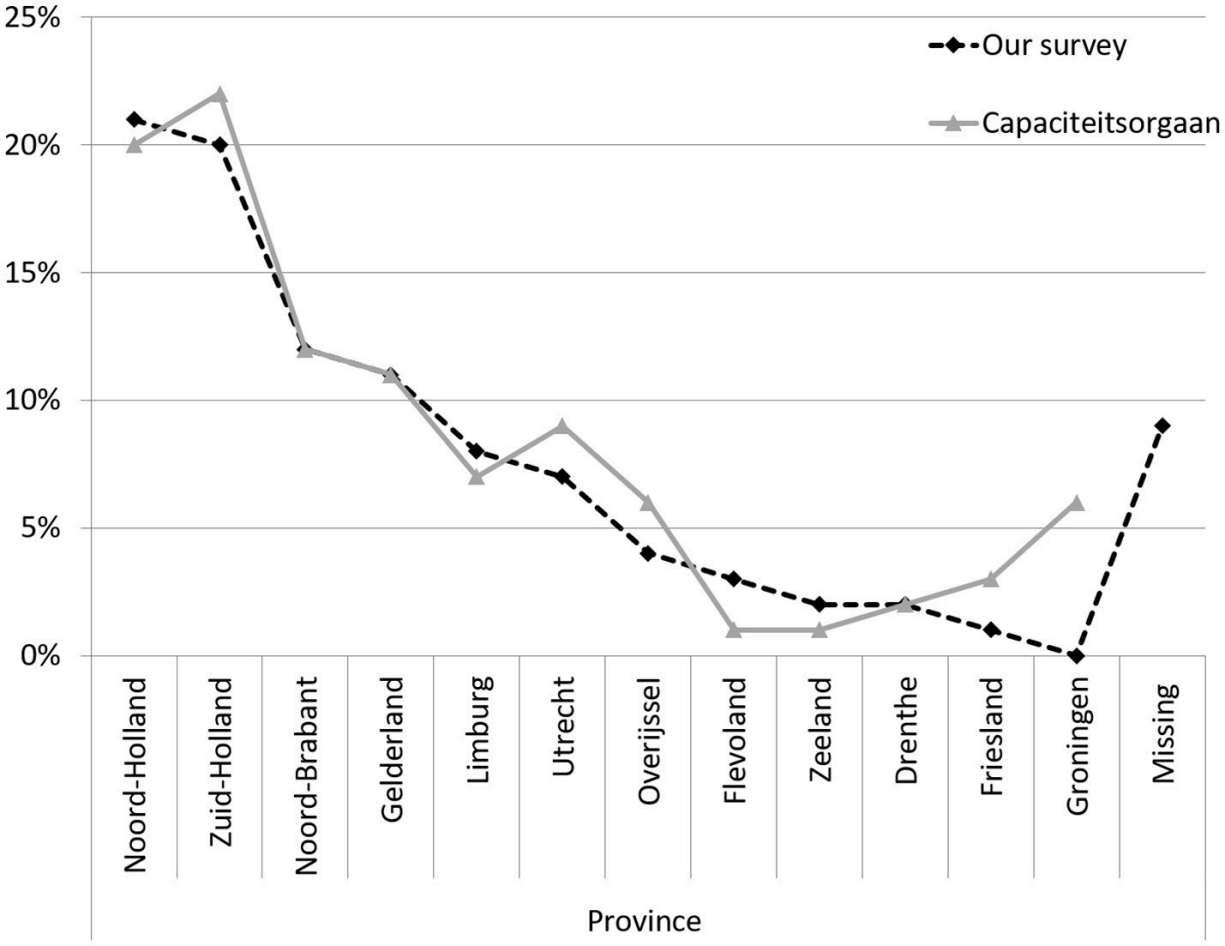
Neurologists and other medical specialists per province, in percentages. Adapted from https://capaciteitsorgaan.nl/app/uploads/2017/04/2017_04_24-DEF-Regionale-spreiding-medisch-specialisten.pdf.

The demographics of the respondents of our survey were consistent with those of the general population of neurologists in the Netherlands (“Nivel survey”) (29). The median age of neurologists in our survey was 44 years (range 39–56.5), compared to 49 years in the Nivel survey. Sixty-two percent was male compared to 72% in the Nivel survey. Figure 1 shows that the distribution of our respondents across the 13 Dutch provinces was similar to that of specialists in general^1^.

### Discussions on Treatment Restrictions: Frequency and Participants

Most consultants in neurology (*n* = 74, 85%) and residents (*n* = 32, 91%) reported to have had discussions on treatment restrictions more than once per 6 months. Thirteen percent (*n* = 14) of the consultants had had one or more discussions per week. Thirty-four (40%) of the neurologists reported that they had had a discussion on treatment restrictions with more than 5 patients over the past 12 months. Of the residents 37% (*n* = 13) had had one or two of these conversations over the past 12 months. Ninety-two percent (*n* = 75) of the consultants in neurology and 65% (*n* = 22) of the residents reported that most of the time these discussions had taken place with the patient and a caregiver, in 8% (*n* = 7) and 35% (*n* = 12), respectively, only with a caregiver, and not once with the patient only. Fifty-five percent (*n* = 44) of the consultants and 53% (*n* = 17) of the residents reported to have had two-tiered discussions on treatment restrictions.

### Discussions on Treatment Restrictions: Timing

**Figure 2:**
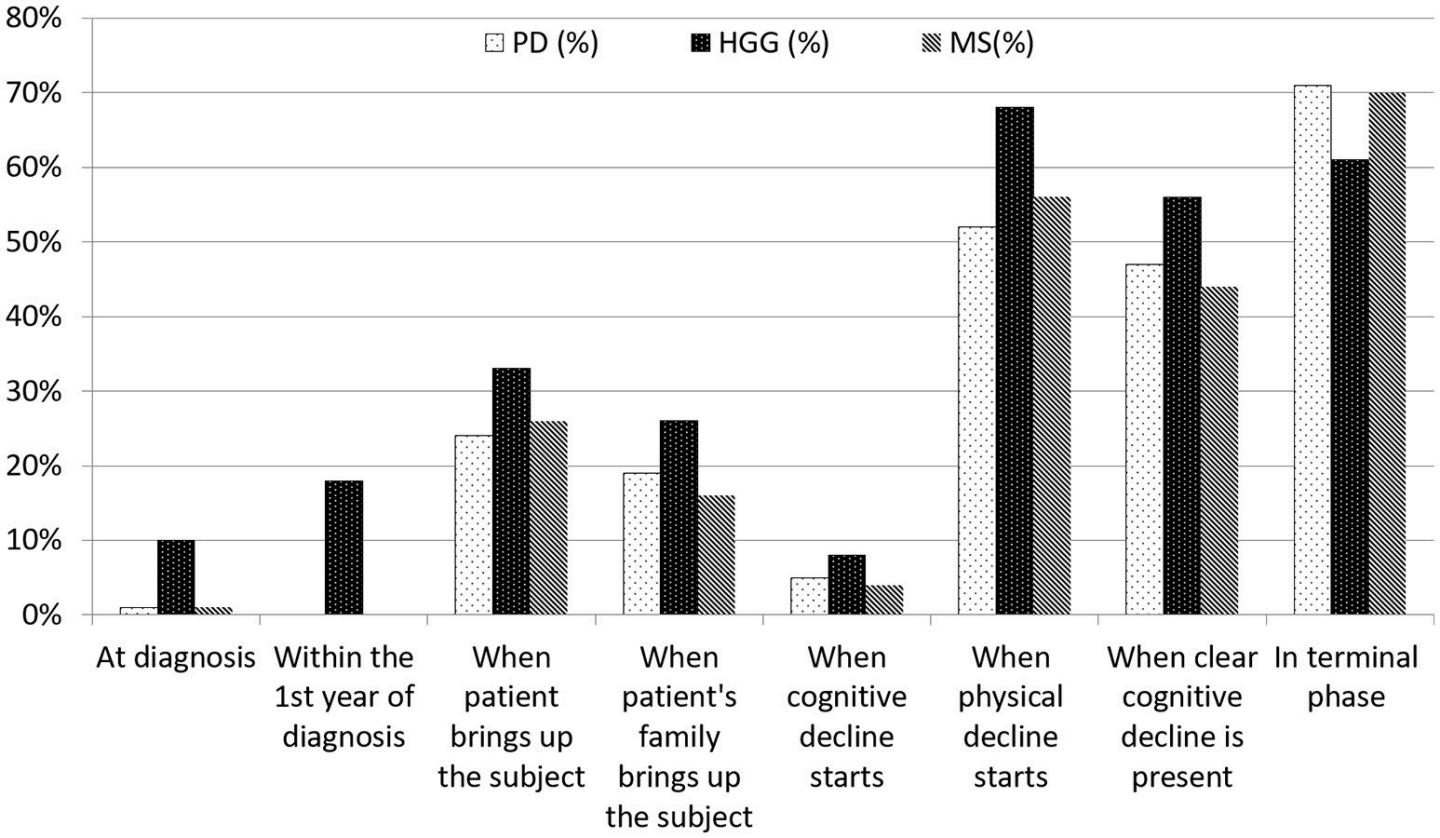
Timing of discussions on treatment restrictions by consultants in neurology and residents. There was no definition provided regarding ‘when cognitive decline starts'. ‘When clear cognitive decline is present' was defined as ‘incapacitating tha patient to (fully) understand and take part in decision-making'.

Eighty-seven percent (*n* = 59) of the consultants in neurology and 92% (*n* = 23) of the residents were of the opinion that a doctor should initiate the discussions. Twenty-four to 33% of our respondents replied that they initiated the discussion “when the patient brings up the subject” and 16–26% “when the patient's family brings up the subject.” In PD and MS, discussions on treatment restrictions took rarely place at diagnosis, and not once within the first year of diagnosis (Figure 2). Seventy-one percent (*n* = 56) of the neurologists and 70% (*n* = 77) of the residents, respectively, discussed treatment considerations in the terminal stages of PD and MS. In HGG, 28% (*n* = 18) of the respondents discussed treatment restrictions within the first year of diagnosis, 68% (*n* = 60) “when physical decline started” and 61% (*n* = 54) in the terminal phase. “The start of cognitive decline” triggered a discussion in 8, 5, and 4% in HGG, PD, and MS, respectively, whereas “when clear cognitive decline had started” led to discussions in 56, 47, and 44% in HGG, PD and MS, respectively (Figure 2).

### Reflections on Discussions on Treatment Restrictions in Recent Cases

Respondents were asked to recall the most recent patient with HGG, PD, or MS with whom they had discussed a treatment restriction. Demographics and diagnosis of respondents' cases are summarized in Table 2. The median time since the discussion had taken place was 1 month (range 1 week−60 months). Sixty percent (*n* = 38) of the patients had cognitive decline and 23% (*n* = 15) were incompetent, of whom 13 (87%) had HGG and 2 (13%) PD. Eighty percent (*n* = 53) of the respondents reported that both patients and caregivers had been present during the discussions. In two instances (3%) the patient was alone, and in 4 (6%) only the caregiver was present. The mean duration of conversations was 29 (SD 13.3) min for neurologists, and 31 min (SD 14.1) for residents.

**Table 2:** Demographics and diagnosis of respondents' cases.

		**Respondents' cases *n* (%)**
Diagnosis	PD HGG MS	16 (24) 43 (65) 7 (11)
Time since diagnosis, in months (median, range)		12 (1 day−20 years)
Age, in years (mean, SD)		65 (15)
Gender (male)		43 (68)

*PD, Parkinson's disease; HGG, high grade glioma; MS, multiple sclerosis*.

There was no consensus on the treatment policy between physician and patient or caregiver/family in 23% (*n* = 15) of the cases for the following reasons: “The patient was not ready to discuss the subject,” “Patients' caregivers were not ready to discuss the subject,” “The patient did not understand why a treatment should be stopped” or “The patient's relatives did not understand why a treatment should be stopped”. In 12 cases (80%) a follow-up appointment was planned, and in 7 cases (47%) the respondent said to have complied with the patient's or relatives' wishes.

### Discussing Treatment Initiation or Withdrawal

Reasons to discuss treatment restrictions varied. “Acceleration of the disease process” was the main reason in 37% (*n* = 22) of the respondents and “Unexpectedly severe functional decline” was mentioned in 10% (*n* = 6). Other reasons included “Exhaustion of the possibilities to favorably influence the disease process” in 28% (*n* = 11) and the “The patient brought up the issue” in 8% (*n* = 5) of the cases. When asked which treatment modalities were considered, respondents could choose “initiate” or “withhold/withdraw”. For patients with PD, resuscitation (*n* = 12, 75%), ventilation (*n* = 12, 75%), admission to intensive care unit (*n* = 12, 75%), and feeding tube (*n* = 10, 63%) were the most discussed issues. In HGG, respondents discussed ventilation (*n* = 34, 79%), resuscitation (*n* = 33, 77%), surgery (*n* = 32, 74%), and admission to the intensive care unit (*n* = 31, 72%). In MS, disease-specific medication was discussed in five instances (71%), non-disease specific medication in four (57%) (i.e., medication for urine incontinence or anti-depressants) and admission to hospital in four (57%).

Table 3 shows the discussions of treatment modalities (initiate or withhold/withdraw) in the specific disease groups in percentages.

**Table 3:** Considered treatment options per disease (percentages).

	**PD *n* = 16** **(*n* (%) initiate | *n* (%) withhold/withdraw)**	**HGG *n* = 43** **(*n* (%) initiate | *n* (%) withhold/** **withdraw)**	**MS *n* = 7** **(*n* (%) initiate | *n* (%) withhold/** **withdraw)**
Resuscitation	0 | 12 (75)	0 | 33 (77)	0 | 3 (43)
Ventilation	0 | 12 (75)	0 | 34 (79)	1 (14) |2 (29)
Feeding tube	0 | 10 (63)	5 (12) | 18 (42)	1 (14) | 1 (14)
Surgery	0 | 9 (56)	5 (12) | 27 (63)	1 (14) | 1 (14)
Antibiotics	3 (19) |6 (38)	6 (14) | 19 (44)	2 (29) | 1 (14)
Corticosteroids	1 (6) | 7 (44)^*^	13 (30) | 10 (23)	1 (14) | 1 (14)
Admission to hospital	3 (19) | 5 (31)	5 (12) | 13 (30)	2 (29) | 2 (29)
Admission to ICU	0 | 12 (75)	1 (2) | 30 (70)	1 (14) | 2 (29)
Disease specific medication	5 (31) | 4 (25)	10 (23) | 18 (42)	0 | 5 (71)
Non-disease specific medication	5 (31) | 4 (25)	7 (16) | 12 (28)	4 (57) | 0

* *In a small number of responses “corticosteroids” were mentioned as discussed treatment option in PD patients. Perhaps this should be considered an error, since this drug is very unusual in PD*.

When asked which terminal care options were discussed, pain alleviation was mentioned in 70% (*n* = 28) of the cases, alleviation of dyspnea in 55% (*n* = 22), and psychosocial support in 53% (*n* = 21) of the cases. Palliative sedation was discussed in 60% (*n* = 24) of the cases.

When asked if, in retrospect, the respondents would have discussed treatment restrictions earlier in the disease process, 27% (*n* = 17) agreed with this statement. Asked for reasons to postpone discussions the following statements were provided: “The patient could not handle it” (25%, *n* = 4), “I did not want to deprive hope” (19%, *n* = 3), “Lack of suffering of the patient” (19%, *n* = 3) and in one case the neurologist said it would have taken too much time.

Preferred location for terminal care was discussed by 88% (*n* = 36) of the respondents. The option “treatment at home” was mentioned in 42% (*n* = 15) of the cases, and the options “hospice” in 36% (*n* = 13).

### Interpretation of the Meaning of Palliative Care

Respondents were asked “What does palliative care mean in your opinion?” Amongst the 77 neurologists and residents who responded to this question, 48% (*n* = 37) used the word “comfort,” 29% (*n* = 22) considered palliative care as “relief of suffering” and in 17% (*n* = 13) it was coded as “quality of life.” “Terminal phase,” “end-of-life” or “no extension of life” was mentioned in 21% (*n* = 16). Thirteen percent (*n* = 10) of respondents used the description “no cure possible” and 12% (*n* = 9) “symptomatic treatment.” The term “supportive care” was used by 9% (*n* = 7).

### Education in Palliative Care

Sixty-four percent (*n* = 44) of the consultants in neurology and 75% (*n* = 18) of the residents reported that they were neither educated nor trained in discussions on treatment restrictions in chronic progressive neurological disease. Fifty-seven percent (*n* = 39) reported that they felt a need for education. Amongst the 25 consultants and 6 residents who were educated or trained, 14 consultants and 4 residents received this education as undergraduates, 18 consultants, and 3 residents during training, and 12 consultants on-the-job. Twenty-two consultants and 5 residents had had education via interactive lessons, for example a role-play, 13 consultants, and 2 residents had had education by supervision. Twenty one of the educated or trained consultants and 5 residents felt that their education/training had been sufficient for their work in clinical practice.

## Discussion

Our survey indicates that in The Netherlands the timing of discussions on treatment restrictions in patients with three chronic progressive neurological diseases (HGG, PD, and MS) varies considerably. The consultants in neurology and residents who responded to our online survey, reported that these discussions regularly took place in the first year of diagnosis in HGG, and mostly in the terminal phase of PD and MS. In all conditions, significant cognitive decline was the most important trigger for the respondents to advance discussions, followed by physical decline, and the terminal phase.

As the response rate was rather low and selection bias might have taken place the findings of our survey have to be interpreted with caution (see also “strengths and limitations”). Importantly, the results are in line with previously reported findings that discussions on treatment restrictions in chronic progressive neurological diseases most often take place after a sudden decline of patients' condition (12, 26, 30).

In ALS, which is considered a paradigmatic disease for palliative care, rapid motor deterioration often includes bulbar impairment leading to speech impairment (31). According to (best practice) guidelines the imminent communication barrier allows no delay in initiating discussions on patients' expectations, wishes and preferences regarding treatment options/restrictions and end-of-life issues (2). There is also a rapid decline in patients with HGG, and in addition the presence of significant cognitive impairment, delirium, communication difficulties, and loss of consciousness impairs their decision-making capacities (12). Up to 79% of patients with HGG have cognitive impairment before treatment, and more than 50% lack full decision-making capacity 4 months after diagnosis (32, 33). This percentage increases, especially in the last months of life (27). However, initiating ACP from diagnosis onwards is still a matter of debate in this patient group (34). In PD and MS, cognitive impairment is also common. In PD, 60% of patients have dementia after a disease duration of 12 years, preceded by a period of mild cognitive impairment, which can even be present at diagnosis (35, 36). Frequencies of cognitive impairment in patients with MS range from 40 to 75% and can become manifest at all stages and in all subtypes of the disease (37, 38) Importantly, cognitive impairment in MS at time of diagnosis is considered a marker of most aggressive pathology (39). In the first consensus review on the development of palliative care in neurology it is therefore recommended to initiate discussions on future care options and wishes early in the course of chronic progressive neurological diseases, especially when cognitive, and communication impairment are likely to occur (40).

Literature on optimal timing of ACP in chronic progressive neurological diseases is scarce. In ALS, ongoing communication of future (palliative) care from diagnosis onwards is strongly recommended, preferably by a multidisciplinary team (40, 41). However, in practice, even in the follow-up of patients with ALS ACP appears to be regularly delayed or triggered by the occurrence of life-threatening complications (30, 42). It is of note that there is a perceived lack of awareness of advance directives amongst health care professionals, in particular hospital staff, which obviously limits the effectiveness of such documents (43, 44). Advance directives are equally underutilized by patients since a study found that only 30% patients with ALS complete them (7). To support both physicians and ALS patients to be better prepared, the recently published NICE guideline recommends to offer patients with ALS the opportunity to discuss their treatment preferences and concerns about care at the end of life at trigger points such as “at diagnosis,” “if there is a significant change in respiratory function,” or “if interventions such as gastrostomy or non-invasive ventilation are needed” (2). Regarding the timing of the discussions, the guideline also advises to take into account the person's current communication ability, cognitive status, and mental capacity. These recommendations are partly based on interviews with patients or (bereaved) caregivers' views. They want sufficient information to be able to take well-considered decisions, as it gives them a feeling of having choice and control over their treatment (14, 45, 46). Timely discussions on end-of life care, options and preferences, have also been shown to lower anxiety, and distress in ALS patients and their caregivers (44).

In the first guideline of the European Association for Neuro-Oncology for palliative care in adults with glioma ACP is defined as a process which is ‘concerned with […] preferences related to non-treatment decisions or preferred place of death. The guideline stresses that ACP is most effective when it is started in a timely fashion, allowing patients, caregivers, and physicians to proactively address the challenges together during the course of the disease'(12). Indeed, there is growing awareness of the importance to openly communicate about patients' expectations, wishes, and preferences during the entire disease trajectory (34). However, in daily practice ACP in patients with HGG is still closely linked to the terminal phase, concerning both timing and content (47). Up to 40% of patients with HGG seem not to be involved in any end-of-life discussion, and the timing of end-of-life discussions may vary widely (1–140 days) (48). A retrospective study amongst physicians on end-of-life decision-making in patients with HGG showed that important topics were life-prolonging treatment (38%), admission to hospital (49%), palliative sedation (29%), and euthanasia (38%). Treatment was withheld in 29% of patients and concerned medication (antibiotics, dexamethasone), radiotherapy, placement of ventricular drain, and artificial administration of food or fluids (27). In our survey the most discussed topics were “resuscitation,” “invasive ventilation,” and “admission to ICU.” One reason for these differences might be that we tried to avoid focusing on the last phase of life in our survey.

In contrast to both ALS and HGG, PD and MS are slowly progressive diseases with an often fluctuating course, unexpected declines, and gradual accumulation of impairments causing significant unmet needs (10, 49, 50). Recently, a study on preferences of patients with PD for communication about ACP showed that most (but not all) of them want prognosis and treatment information early, and that many expect their healthcare providers to bring up these issues (51). A qualitative study involving patients with PD underlined this: about half of the interviewees wanted their neurologist to raise the subject of ACP as an adjunct to usual care (49). And a survey amongst surrogates of patients in advanced stages of PD indicated that living wills might be completed by up to 94% of the patients, but shared with a physician by only 38% of them (52).

In a survey study on MS patients' palliative care needs the majority of respondents found it important to address the progression of disease and ACP. More than one-third wished to talk about end-of-life issues (53). One study addressing long-term care planning showed that on average only 11% healthcare providers discussed this issue, ranging from 10 to 26% for mildly affected and severely affected patients, respectively (54).

Currently, there are efforts being made to incorporate palliative care principles in PD and MS patients' long-term follow-up (1, 7, 8, 40). In line with that, the use of triggers to identify significant deterioration has been suggested, and end-of-life care needs are being mapped (15, 55). Pertinent topics to discuss in advanced PD and MS should include tube feeding, the use of antibiotics in case of infection, non-invasive ventilation in case of respiratory failure, and resuscitation (56).

In our survey, the most discussed treatment options with PD patients or their caregivers were “resuscitation,” “invasive ventilation,” “admission to ICU,” and “use of a feeding tube.” In MS the issues of “disease-specific medication” and “non-disease specific medication” and “admission to hospital” were most frequently discussed. Due to the small sample size (PD = 16, MS = 7) it is not possible to draw any conclusions about this discrepancy.

The results of our survey suggest that in most cases the consultants in neurology decided on the timing of discussions on treatment restrictions, and indeed the respondents were of the opinion that a doctor should initiate these conversations. However, they also appeared to be sensitive to the wishes of the patient or the patient's family if they brought up the subject. Uncertainty about optimal timing often causes postponement of discussions on treatment restrictions (57). In our survey reasons to postpone discussions included “I did not want to deprive hope” and “The patient could not handle it.” A “wait and see policy” concerning discussions about the appropriate amount of future care seems to be a quite common strategy of many healthcare professionals (58–63).

At the end of our survey, we asked via an open-ended question what “palliative care” meant to the participating consultants in neurology and residents. There was a great variety of responses of which the terms “comfort,” “quality of life,” “end-of-life care,” and “terminal care” were mentioned most frequently. This is consistent with previous research amongst health care professionals, including neurologists (1). One common misconception is that discussions on future (palliative) care may signal the ‘beginning of the end', despite the finding that usual neurological care during follow-up of patients with chronic progressive diseases can go hand in hand with palliative care, including ACP (9, 64). The term “palliative care” is not only confusing for neurologists, but also for patients who might not be interested in “palliative care,” but willing to attend a team-based clinic providing intensive symptom management and psychosocial support (15). Therefore, some clinicians suggest to talk about “supportive care” (65). In our survey 7% of the consultants in neurology associated palliative care with supportive care.

In our study, most neurologists reported that they were experienced in having discussions on treatment restrictions, i.e., not initiating or withdrawing treatment. However, when asked about their education 66% of the respondents reported that they had not been educated or trained in having these conversations, and about half of them indicated that they felt a need for education on this topic. Those who were trained felt that it was sufficient for daily practice. Various authors have described a general lack of education in palliative care skills amongst physicians, residents, and students (15, 66–70). A recent study investigating the effectiveness of training in palliative and end-of-life communication skills in medical students showed that nearly 80% indicated retention of communication skills after 1 year with regard to “giving bad news,” followed by “talking about death and dying,” and “end-of-life preferences/do not resuscitate” in 40–45% of the students (71). Overall, there is quite some evidence that communication training improves discussions on diagnosis, treatment options, and preferences including end-of-life care as experienced by both healthcare professionals and patients with progressive diseases and their caregivers (16, 72–76). Interestingly, a lack of an empathic response was noted as a gap in the neurologists' skills by patients and caregivers (14).

### Strengths and Limitations

To the best of our knowledge our survey is the first addressing the daily practice of consultants in neurology and residents in the Netherlands concerning discussions on treatment restrictions in patients with PD and MS. We found one study in which physicians and bereaved relatives were questioned about decision-making and end-of-life practices in patients with HGG (27).

The overall response rate to our survey was 15%, which is rather low. However, we assume that this still represents a fair proportion of the neurological practices since in most hospitals subspecialized neurologists care for patients with HGG, PD, or MS, respectively. Additionally, the relatively low response rate may be explained by the distribution of the questionnaire via the secretariat of the neurology department for privacy reasons. There are other limitations. First, there may have been “self-selection bias.” The majority of respondents had a specialization area so we cannot exclude that consultants in neurology and residents with a special affinity with the topic filled in the questionnaire. Second, due to privacy reasons we do not have information about the non-respondents. Third, we might have influenced the respondents' views. As we aimed to study discussions on treatment restrictions during the whole disease process, we cautiously avoided to use the terms “palliative care” and “end-of-life care” in the questionnaire. Still, it may well be that the phrasing of our questionnaire has triggered certain associations given the responses suggesting that discussing treatment restrictions was closely linked to (starting) end-of-life care. Finally, our data concerning the content of discussions are more representative of HGG than PD and MS due to unequal response rates.

In conclusion, our study suggests that discussions on initiating, withholding, or withdrawing treatment in patients with HGG, PD, and MS are mainly determined by significant cognitive and physical deterioration or the imminent terminal phase. Thus, they usually take place at advanced stages of the disease. The reasons are multilayer and changing daily practice will be a complex challenge. However, education in palliative care skills and knowledge of the overall interest of patients to be involved may be an important step to improve daily clinical practice.

Concrete future research projects arising from our findings should specifically investigate patients' wishes and preferences regarding timing and content of discussions about future care options.

## Author Contributions

HAWW contributed in analyzing the data, interpretation of the data, writing, and revising of the manuscript, and accepts responsibility for the corresponding author. AAS contributed to the study design, data collection, interpretation of the data, and writing and revising of the manuscript. DLW contributed to the study design, interpretation of the data, and writing and revising of the manuscript. MdV contributed to the study design, interpretation of the data, and writing and revising of the manuscript. All authors are in agreement with the contents of the manuscript and provide approval for publication of the content.

### Conflict of Interest Statement

The authors declare that the research was conducted in the absence of any commercial or financial relationships that could be construed as a potential conflict of interest.

## Footnotes

^1^Available online at: https://capaciteitsorgaan.nl/app/uploads/2017/04/2017_04_24-DEF-Regionale-spreiding-medisch-specialisten.pdf
